# Epidemiology of Orthopaedic Trauma Cases at a Tertiary Level Hospital in Nepal: An Observational Study

**DOI:** 10.31729/jnma.9152

**Published:** 2025-07-31

**Authors:** Manoj Kandel, Sarik Kumar Shrestha, Krishna Prasad Paudel, Rabin Bom, Ashmita Paudel, Toya Raj Bhatta, Pritam Chaudhary, Prakash Kandel, Sumit Sharma

**Affiliations:** 1 Department of Orthopaedics and Trauma Surgery, Bharatpur Hospital, Chitwan, Nepal; 2 Department of Emergency Medicine, Bharatpur Hospital, Chitwan, Nepal; 3 Department of Critical Care Medicine, Bharatpur Hospital, Kathmandu, Nepal; 4 Department of Orthopaedics and Trauma Surgery, Patan Academy of Health Sciences, Lalitpur, Nepal; 5 Department of Orthopaedics and Trauma Surgery, Bakulahar Ratnanagar Hospital, Chitwan, Nepal.

**Keywords:** *epidemiology*, *injury*, *physical trauma*, *trauma*

## Abstract

**Introduction:**

Trauma is a leading cause of morbidity and mortality worldwide. While there has been significant progress in understanding trauma in high-income countries, data from Nepal is limited. This study's main objective was to explore the demographic profile, injury patterns, and treatment of orthopaedic trauma patients presenting to a tertiary-level hospital in Nepal.

**Methods:**

This was a retrospective study conducted at a tertiary-level hospital of Nepal. All complete records of trauma cases presenting to the hospital from January 2017 to December 2022 were included in the study. Data were collected from electronic and manual records, anonymized, and analyzed with Microsoft Excel 2021.

**Results:**

A total of 27,893 complete records were included in the study. Of these, 19,679 (70.55%) patients were male. Patients aged 30-44 years accounted for 9,566 (34.29%) cases. The mechanisms of injury included falls in 12,585 (45.12%) cases and road traffic accidents in 8,524 (30.56%) cases. Fractures were identified in 17,593 (63.07%) patients, with 9,800 cases classified as lower extremity fractures. Additionally, 18,595 (66.67%) patients were referred from outside the district.

**Conclusions:**

Orthopaedic trauma was seen more commonly in males and the most common causes of injury are falls and RTA. Fractures were the most common pattern of orthopaedic injuries and lower extremity fractures were the most common.

## INTRODUCTION

Trauma is a leading cause of morbidity and mortality worldwide.^1,2^ The impact is more severe in low- and middle-income countries (LMICs), where injury prevention programs and healthcare resources are lacking.^3-5^

There have been a few studies conducted in Nepal focusing on the epidemiology of RTA cases and orthopaedic admissions.^6,7^ However, the epidemiology of trauma cases may differ according to the geographic location. Therefore, there exists a research gap in understanding the trauma epidemiology in Nepal as a whole.

This gap can only be filled if further studies on the epidemiology of trauma cases are conducted in different places of Nepal.

This study's main objective was to explore the demographic profile, injury patterns, and treatment of trauma cases at a tertiary-level hospital in Nepal. This study also aims to add to the information given by previous studies from Nepal to better understand the trauma epidemiology in Nepal across different regions.

## METHODS

This was a retrospective study conducted at a tertiary-level hospital of Chitwan, Nepal utilizing available data of four years from 1^st^ January, 2017 to 31^st^ December, 2022. Ethical approval for the study was obtained from the Institutional Review Committee (IRC) (Reference no: 079/80-016).

The study included all orthopaedic trauma cases registered in the record book of the ED from 1^st^ January, 2017 to 31^st^ December, 2022. Only dislocations, tendon injuries, and ligament tears that were clearly diagnosed and recorded at presentation were included. Many of these injuries need imaging or follow-up to confirm and may have been missed. When noted, they were grouped under “soft tissue injuries” or “fracture-dislocations.” Thus, the study focuses more on fracture cases, due to the nature of emergency records available. The cases with incomplete or missing records, patients who were brought dead were excluded. The observed sample comprised all the orthopaedic trauma cases presenting to the Emergency Department (ED) during the above period. Since this was an epidemiological study to recognize the pattern of injuries and outcomes, total population sampling was done. All cases meeting the inclusion criteria were included in the study.

Data were collected from the hospital’s electronic medical records (EMRs) and manually recorded logs. The details of admitted patients were available from the EMRs while the details of patients discharged after treatment from the ED had to be collected from the manually recorded logs. Data was extracted by the investigators. All the complete records of trauma cases available within the study period were included in the study The key data extracted from the records included demographic details, injury details (type of injury, injury pattern, mechanism of injury), diagnosis, and treatment. Collected data were anonymized and stored securely. Each patient was assigned a unique identifier to ensure confidentiality. Calculations were performed using Microsoft Excel 2021 software. Descriptive statistics were used to summarize the data. Categorical variables were presented as frequencies and percentages, and continuous variables as means and standard deviations.

## RESULTS

A total of 32,139 trauma patients were registered in the Emergency Department of Bharatpur Hospital during the study period. After excluding 4,246 incomplete records, 27,893 complete records were included in the analysis. Of these, 19,679 (70.55%) patients were male and 8,214 (29.45%) were female. Patients in the 30-44 years age group accounted for 9,566 (34.29%) cases. Fall injuries were reported in 12,585 (45.12%) cases, and road traffic accidents in 8,524 (30.56%) cases.

Fractures were identified in 17,593 (63.07%) patients. Among these, closed fractures were recorded in 13,303 (75.62%) cases and open fractures in 4,290 (24.38%) cases. The mechanism of injury included falls accounting for 12,585 (45.12%) cases, followed by road traffic accidents with 8,524 (30.56%) cases. Soft tissue injuries, including tendon and ligament injuries, accounted for 10,300 (36.93%) cases. Dislocations were categorized under fracture-dislocations of the spine, or excluded if not clearly noted. The figures represent consistent data from the emergency setting and may not capture the entire spectrum of orthopaedic trauma. (Table 1).

**Table 1:** Demography and mechanism of injury among included cases (n=27,893).

Characteristics	n(%)
Sex	
Male	19,679(70.55)
Female	8,214(29.45)
Age group	
0-14 years	1,312(4.70)
15-29 years	7,510(26.92)
30-44 years	9,566(34.29)
45-59 years	6,867(24.63)
>60 years	2,638(9.46)
Mechanism of injury	
Fall	12,585(45.12)
Road traffic accident (RTA)	8,524(30.56)
Physical assault	3,476(12.46)
Work-related injuries	2,296(8.23)
Sports injuries	1,012(3.63)
Diagnosis	
Fractures	17,593(63.07)
Soft tissue injuries	10,300(36.93)

**Table 2:** Distribution of upper and lower extremity fractures by bone involvement (n=27,893).

Fracture location	n(%)
Upper Extremity Fractures	
Radius and ulna	3,217(46.32)
Humerus	1,227(17.67)
Carpal bones	1,697(24.44)
Clavicle	804(11.57)
Lower Extremity Fractures	
Femur	4,250(43.37)
Tibia	3,490(35.61)
Fibula	1,400(14.28)
Pelvis and acetabulum	660(6.74)

**Table 3:** Distribution of vertebral fractures by site (n=848).

Fracture type	n(%)
Dorso-lumbar spine	
Compression fracture	350(66.29)
Burst fracture	128(24.24)
Fracture-dislocation	50(9.47)
Cervical spine	
Simple fracture (e.g., spinous process fracture)	125(39.06)
Fracture-dislocation	95(29.69)
Severe fracture (e.g., Jefferson fracture, Hangman’s fracture)	100(31.25)

**Table 4:** Surgical interventions for fracture stabilization (n=5,333).

Surgical intervention	n(%)
Open Reduction and Internal Fixation (ORIF) with plating	
Femoral fractures	1,025(19.22)
Tibial fractures	853(15.99)
Upper extremity fractures (radius, ulna, humerus)	1,300(24.38)
Closed Reduction and Internal Fixation (CRIF)	
Femoral fractures	950(17.81)
Tibial fractures	875(16.41)
Spinal instrumentation	
Dorso-lumbar spine fractures	210(3.94)
Cervical spine fractures	120(2.25)

Among the 6,945 upper extremity fractures, radius and ulna fractures accounted for 3,217 (46.32%) cases, followed by carpal bone fractures with 1,697 (24.44%) cases. In the 9,800 lower extremity fractures, there were 4,250 (43.37%) femur fractures followed by 3,490 (35.61%) tibia fractures reported (Table 2).

Among 528 dorso-lumbar spine fractures, compression fractures accounted for 350 (66.29%) cases and burst fractures for 128 (24.24%) cases. In 320 cervical spine fractures, simple fractures (such as spinous process fractures) represented 125 (39.06%) cases, fracture-dislocations 95 (29.69%) cases, and severe fractures (including Jefferson and Hangman’s fractures) 100 (31.25%) cases (Table 3).

During the study period, a total of 5,333 surgical procedures were performed at the hospital to manage displaced or unstable fractures. These surgeries were conducted either on an emergency basis or following admission to the ward. Open reduction and internal fixation (ORIF) with plating was carried out in 1,025 (19.22%) femoral fractures, 853 (15.99%) tibial fractures, and 1,300 (24.38%) upper extremity fractures involving the radius, ulna, or humerus. Closed reduction and internal fixation (CRIF) was performed in 950 (17.81%) femoral fractures and 875 (16.41%) tibial fractures. Spinal instrumentation was used in 210 (3.94%) dorso-lumbar spine fractures and 120 (2.25%) cervical spine fractures (Table 4).

Throughout the study period, external fixators were applied in 204 open femoral fractures and 402 open tibial fractures.

Regarding referral patterns, 18,595 (66.67%) trauma patients arrived from outside the district, while 9,298 (33.33%) were from within the district. Patients were referred from all 77 districts of Nepal, with cases originating notably from Bagmati Province and Gandaki Province.

## DISCUSSION

The demographic findings of this study reveal that trauma incidents predominantly affect men, with males accounting for over two-thirds of cases, a trend observed in similar studies across South Asia and other LMICs. Previous research studies conducted in Nepal also aligns with this result, demonstrating a consistent male predominance in trauma cases, including road traffic accidents (RTAs) and fall injuries.^6,7^ We found men to be more often involved in trauma cases compared to women. Other studies from Nepal such as those conducted in Kathmandu, Lalitpur, Hetauda and Karnali also showed greater involvement of males in trauma.^8-11^ Another nation-wide study conducted in 15 districts also showed males to be more commonly involved in trauma.^12^ Studies from other countries also show similar results. A study from Australia also showed that males are more likely to be involved in trauma.^13^ This gender disparity could be influenced by various socio-cultural factors, including the roles and activities that men engage in, which are often riskier compared to those of women. The greater tendency of taking risks, greater participation in outdoor activities, greater involvement in manual labour, and greater percentage of drivers and motorcycle riders being males are some of the reasons for higher incidence of trauma in males.^14^ However, studies from England and China have shown equal sex distribution among trauma patients.^15-17^ This may be because of greater number of older patients included in these studies as osteoporotic fractures from low-energy trauma are more common in females.^15^

The majority of the trauma patients fell within the 15-44 years age group, similar to other studies from Lalitpur, Hetauda, Karnali and Dhulikhel.^9-11,18^ Similar results were seen in studies from other developing countries such as India, Saudi Arabia, Mozambique.^19-23^ Similar age distribution was seen in a study from Iran as well.^14^

The most common mechanism of injury in this study was fall injury, which includes fall from height and low-energy falls. Falls were the most common mechanism of injury in studies from Kathmandu, Lalitpur and Karnali.^8,9,11^ Fall injuries are particularly prevalent among the elderly and children, with similar findings reported in Nepal as well as neighbouring countries such as India and Pakistan.^24-26^ Research from these countries shows that falls are the leading cause of injuries, especially among those below 15 years of age.^24-26^ A study from Iran also identified falls as the most common mechanism of injury.^14^ However, a study from Hetauda showed RTA to be the most common mechanism of injury.^10^ Other studies from India and Nigeria also showed that RTAs are the commonest cause of injury.^19,27^ The high incidence of falls in Nepal could be attributed to environmental and socio-economic factors, such as poor infrastructure, inadequate housing conditions, and lack of awareness about fall prevention, particularly in vulnerable groups like children and the elderly.

Road traffic accidents (RTAs) were the second most common cause of trauma in this study, a finding that is consistent with previous studies conducted in Nepal.^6,7-9,11^ Another study also reported that RTAs, particularly motorcycle accidents, are the most common form of injury, especially in urban areas and among individuals aged 15-40 years.^28^ A study from Hetauda also showed that young adults are more commonly involved in RTAs.^10^ However, due to the limitations of data availability in our study, we could not analyse specific types of vehicles involved in RTAs. However, a study from Kathmandu had shown that two-wheelers are more commonly involved in RTAs than four-wheelers.^29^ This is an important consideration for future studies, as motorcycle-related accidents are becoming increasingly prevalent in urban areas across Nepal and other South Asian countries.

Physical assaults, including domestic violence, group fights, and workplace altercations, accounted for about 12.46% of the trauma cases in this study. Similar results were seen in studies from Kathmandu and Karnali.^8,11^ These injuries were present across all age groups, and while the victims of domestic violence were mostly women, men were more frequently assaulted by unspecified individuals which aligns with the findings from other studies.^30^ The high prevalence of domestic violence and physical assault calls for stronger community health programs focused on violence prevention and awareness.

Work-related injuries were another common category of trauma, with cut injuries and crush injuries being the most frequent in our study. Work-related injuries are a known issue in industrial and construction sectors in Nepal, and similar findings have been reported by other studies.^8,30,31^

The study revealed that the commonest diagnosis in orthopaedic trauma patients was of a fracture, which is similar to the results of studies conducted in India and China.^19,32^ However, another study from Karnali, Nepal found soft tissue injuries to be more common.^11^ This difference may be due to the fact that our center is a referral center receiving high number of fracture cases referred from other hospitals. In this study, closed fractures were seen in 75.62% cases and open fractures were present in 24.38% cases. Similar results were seen in other studies as well. A study from India showed that open fractures are present in 28.34% cases.^18^ Another study from Nepal showed that open fractures were present in 31.6%.^33^

This study mostly highlights fracture-related trauma, as soft tissue injuries and dislocations were not always properly recorded. These injuries are harder to diagnose at first visit and may need follow-up or imaging. As a result, the study may underreport them, but it still offers useful insights into the patterns of trauma that can be tracked well in emergency data. Future research should include OPD or surgical records for a fuller picture.

Regarding injury location, lower extremity fractures were the most prevalent among all fractures, followed by upper extremity fractures. This was similar to the findings of other studies from Nepal.^9-11,18^ Studies done in India, China and Iran also showed that fractures of the lower extremity were the most commonly seen fractures followed by upper extremity fractures.^19,32,34^ Some studies have however identified upper extremity fractures such as distal radius fracture to be the most common.^14^ This is likely due to higher involvement of patients with osteoporosis and fractures resulting from low-energy trauma in these studies.

Throughout the study period, a substantial number of fracture stabilization surgeries were performed at the hospital to manage displaced or unstable fractures. The most common surgical procedure performed was ORIF followed by CRIF and spinal instrumentation. We did not find any study on epidemiology of trauma cases from Nepal that provide details of surgeries done for definitive management of fractures in trauma cases during our literature search.

This study adds to the pre-existing literature on the epidemiology of trauma cases in different places of Nepal. This study further helps to understand the similarity and differences in the epidemiology of trauma cases in different locations of the country. The findings of this study provide a comprehensive overview of the epidemiology of orthopaedic trauma cases presenting to a tertiary care centre in Chitwan, Nepal. The high prevalence of trauma, particularly among males and young adults, is consistent with global trends and highlights the significant burden of trauma in low- and middle-income countries. Trauma care in Nepal, particularly in the ED, requires strategic planning, better resource allocation, and enhanced trauma prevention programs. The high burden of RTAs highlights the need for improved road safety measures, including better infrastructure, stricter enforcement of traffic laws, and public awareness campaigns. The prevalence of falls among vulnerable populations such as the elderly and children call for targeted prevention programs, including home safety assessments and community education.

However, there are a few limitations of this study. Firstly, this study is limited by its retrospective design and reliance on existing medical records, which may be subject to documentation errors. Additionally, the study was conducted at a single tertiary care centre, which may limit the generalizability of the findings to other settings. Additionally, we did not include long-term follow-up data of the patients included in the study. Future research should include prospective multi-centre studies and also include follow-up to assess long-term outcomes and rehabilitation to provide a more comprehensive picture of trauma epidemiology in Nepal. Also, cases of soft tissue injuries were discharged from the Emergency Department and further records of later follow-up were not available in records. Hence, we could not include the definitive diagnosis of these cases such as ligament injuries which might have been made during follow-up visits in the outpatient department.

## CONCLUSION

Orthopaedic trauma is seen more commonly in males and the most common causes of injury are falls and RTA. Fractures were the most common pattern of orthopaedic injuries and lower extremity fractures were more common. Research into appropriate strategies for prevention of injuries is required and should start with the establishment of institutional and regional trauma registries for complete documentation of relevant data.

Although fractures were most clearly captured, dislocations and soft tissue injuries are also important and likely undercounted in emergency records. For a better understanding of orthopaedic trauma in Nepal, more complete trauma registries and follow-up systems are needed. This study still reflects a major portion of trauma patterns seen in real-world practice and highlights the need for better injury reporting systems.

## Data Availability

The data are available from the corresponding author upon reasonable request.
